# Exome sequencing of multiple-sclerosis patients and their unaffected first-degree relatives

**DOI:** 10.1186/s13104-017-3072-0

**Published:** 2017-12-12

**Authors:** Sheila Garcia-Rosa, Maria Galli de Amorim, Renan Valieris, Vanessa Daccach Marques, Julio Cesar Cetrulo Lorenzi, Vania Balardin Toller, Guilherme Sciascia do Olival, Wilson Araújo da Silva Júnior, Israel Tojal da Silva, Amilton Antunes Barreira, Diana Noronha Nunes, Emmanuel Dias-Neto

**Affiliations:** 1 Lab. of Medical Genomics, International Research Center, A.C.Camargo Cancer Center, Rua Taguá 440, 1st Floor, São Paulo, SP 01508-010 Brazil; 2 Laboratory of Computational Biology and Bioinformatics, International Research Center, A.C.Camargo Cancer Center, Rua Taguá 440, 1st Floor, São Paulo, SP 01508-010 Brazil; 3 Department of Neurosciences, Clinical Neuroimmunology Division, Medical School and Hospital das Clínicas of Ribeirão Preto, University of São Paulo (USP), Avenida Bandeirantes, 3900, Ribeirão Preto, SP 14049-900 Brazil; 4 Center for Medical Genomics, HCFMRP/USP, Avenida Bandeirantes, 3900, Ribeirão Preto, SP 14049-900 Brazil; 5 Department of Genetics, Ribeirão Preto Medical School, University of São Paulo (USP), Avenida Bandeirantes, 3900, Ribeirão Preto, SP 14049-900 Brazil; 6 Neurosciences Research Group, Faculdade de Ciências Médicas da Santa Casa de São Paulo, Rua Doutor Cesário Motta Júnior, 61 - Vila Buarque, São Paulo, SP 01221-020 Brazil; 7 Lab. of Neurosciences (LIM-27), Institute of Psychiatry, Faculdade de Medicina, Universidade de São Paulo, São Paulo, SP Brazil

**Keywords:** Whole exome sequencing, Inheritance, ‘de novo’, Remittent-recurrent, Multiple sclerosis

## Abstract

**Objectives:**

The understanding of complex multifactorial diseases requires the availability of a variety of data for a large-number of affected individuals. In this data note here we provide whole exome sequencing data from a set of non-familiar multiple-sclerosis (MS) patients as well as their unaffected first-degree relatives. This data might help the identification of genomic alterations, including single nucleotide polymorphisms, de novo variations and structural genomic variations, such as copy-number alterations that may impact this disease.

**Data description:**

This dataset comprises the full exome of 28 Brazilian subjects grouped in eight distinct families, consisting of four complete trios (mother–patient–father) plus another four complete trios with one added unaffected sibling. In total, we present the full exome data of eight patients diagnosed with recurrent remittent multiple sclerosis. Diagnoses were made by experienced neurologists and all enrolled patients had at least 5 years of follow up and specific MS treatment. Exomes were sequenced from leukocyte-derived DNA, after the capture of exons using biotinylated probes, in the Ion Proton platform. For each exome we generated an average of 66.1 million good quality mapped reads with an average length of ~ 160nt. On average, for 90% of the exome a vertical coverage above 20× was reached.

## Objective

The understanding of complex multifactorial diseases requires the availability of information for thousands of affected individuals. The release of this data is an attempt to contribute to studies of remittent recurrent multiple-sclerosis (RRMS). The sequencing data presented here was generated to help the identification of genomic variants located in the exomes of RRMS patients from Brazil, a country with elevated ethnic admixture [1–3]. Exomes of the patient’s first-degree unaffected relatives (both parents and siblings) were also sequenced to allow the study of ‘de novo’ variations which, so far, have been under investigated in RRMS. The exome variations observed here may help the study of relevant single nucleotide polymorphisms, de novo- and structural genomic variations—such as copy-number alterations that may be associated with the disease [4–8]. In total, 28 individuals have been sequenced including eight RRMS patients as well as a set of another 20 unaffected first-degree relatives. Some variations suggested by these data have been validated by Sanger sequencing and subjected to functional analysis, have been presented in a manuscript that is currently under review (Garcia-Rosa, et al. unpublished). Most of the exomes presented here have not been explored yet, but it is certain that the whole dataset can be useful for subsequent studies by other groups interested in this field.

## Data description

This study was focused on families containing one member diagnosed with RRMS, with both biological parents alive, and no description of other cases of neurodegenerative or neuropsychiatric cases in the family. Subjects were diagnosed as RRMS patients by experienced neurologists, using currently accepted protocols [9]; paternity and maternity were confirmed for all families using the Variant Call Format (VCFs) files to verify the compatibility status of single nucleotide polymorphisms for all patients and their siblings, according to their status in the exomes of the parents [10]. The study comprised a total of 28 Brazilian individuals, divided in eight families. The trio mother–patient–father was available for all eight families and four of the families also included one unaffected sibling. According to this, subjects were designated in familial groups (G) from G1 to G8. For each family group (G), individuals were classified with single letters designating the mother (M), the father (F) and the patient (P), as well as the unaffected patient–brother (B) or the unaffected patient–sister (S). Therefore, G1F indicates the exome sequence of the father of group 1 and G5S indicates the exome sequence of the unaffected sister of the patient of family 5, and so forth. For each subject, genomic DNA was obtained from leukocytes isolated from 4 mL samples of peripheral whole blood using the Wizard Genomic DNA purification kit (Promega, USA). One microgram of genomic DNA samples from eight affected individuals (G1P to G8P), their parents (16 samples: G1 M to G8 M for mothers and G1F to G8F for fathers) as well as four siblings (G2B, G4S, G5S and G6S) were prepared for WES (whole exome sequencing) using the Ion TargetSeq Exome Capture Kit (Thermo Fisher, USA). For this, the DNA was fragmented for 30 min (Ion Shear Plus Enzyme Mix II), adapters and barcodes were ligated and size selected (275–295 bp). Libraries were amplified (8 cycles) using the Platinum PCR Super Mix High Fidelity. Exons were captured from 500 ng of amplified libraries by hybridization using biotinylated probes (Ion-TargetSeq-Exome-50 Mb-hg19_RevA), following the manufacturer’s instructions. This panel covers a total of 46.2 million bases, encompassing 25,313 genes and 267,049 targets. The instructions to access and to download the corresponding target regions (.bed files) of this specific panel are provided by the manufacturer [11].

WES libraries were sequenced using the Ion PI Sequencing 200 kit V3 in the Ion Proton sequencing platform (CIPE, A.C.Camargo Cancer Center, São Paulo, SP, Brazil). On average, each sequencing run in a P1 chip generated about 10–12 billion mapped bases. Sequencing reads were mapped against the specific exome target region (Ion-TargetSeq-Exome-50 Mb-hg19_revA), using the configuration TargetSeq—Proton—Germ Line—High Stringency, and the Torrent Suite V4.2.1. For each individual exome we generated an average of 66.1 million good quality mapped reads with an average length of ~ 160 nt. On average, for 90% of the exome we reached a vertical coverage of at least 20×. The data described in the present report has not been filtered prior to deposition in the short reads archive (/sra) of NCBI, therefore, mapped and unmapped reads are available through the provided links shown in Table 1.

**Table 1:** Overview of data files/data sets

Label	Name of data file	File types (file extension)	Data repository and identifier (DOI or accession number)	License
Data file 1	SAMN07947504–G1M	Binary sequence alignment/map file (.bam)	Sequence Read Archive http://www.ncbi.nlm.nih.gov/sra (SRP122913)	CC-BY
Data file 2	SAMN07947505–G1P			
Data file 3	SAMN07947506–G1F			
Data file 4	SAMN07947507–G2B			
Data file 5	SAMN07947508–G2M			
Data file 6	SAMN07947509–G2P			
Data file 7	SAMN07947510–G2F			
Data file 8	SAMN07947511–G3M			
Data file 9	SAMN07947512–G3P			
Data file 10	SAMN07947513–G3F			
Data file 11	SAMN07947514–G4S			
Data file 12	SAMN07947515–G4M			
Data file 13	SAMN07947516–G4P			
Data file 14	SAMN07947517–G4F			
Data file 15	SAMN07947518–G5S			
Data file 16	SAMN07947519–G5M			
Data file 17	SAMN07947520–G5P			
Data file 18	SAMN07947521–G5F			
Data file 19	SAMN07947522–G6S			
Data file 20	SAMN07947523–G6M			
Data file 21	SAMN07947524–G6P			
Data file 22	SAMN07947525–G6F			
Data file 23	SAMN07947526–G7M			
Data file 24	SAMN07947527–G7P			
Data file 25	SAMN07947528–G7F			
Data file 26	SAMN07947529–G8M			
Data file 27	SAMN07947530–G8P			
Data file 28	SAMN07947531–G8F			

## Limitations

The sequencing platform used here generates high quality reads, but homopolymeric regions—the consecutive repetition of the same nucleotide for five times or more—have been shown to contain higher error rates that affect the length of the repetitive unit [12]. Previous work recommended that variants located in the junction of two homopolymers, as well as variants that indicate the elongation (insertions) or the shortening (deletions) of homopolymeric repeats, should be confirmed by Sanger sequencing [12]. Therefore, variants in homopolymeric regions identified in our data should be evaluated with extra caution. Compared to other Next Generation Sequencing platforms, such as Illumina, the platform used here presents higher error rates in variants identified as small insertions and deletions (InDels). Also, as the number of individuals sequenced here is small, investigators should seek more data and experimental validation to help identifying genomic alterations that might be associated with RRMS.

## Abbreviations

RRMS: remittent recurrent multiple sclerosis
WES: whole exome sequencing
VCF: variant call format
InDels: insertions and deletions (InDels)
